# 1-Pamitoyl-2-Linoleoyl-3-Acetyl-rac-Glycerol May Reduce Incidence of Gemcitabine-Induced Neutropenia: A Pilot Case-Controlled Study

**DOI:** 10.14740/wjon937e

**Published:** 2015-08-27

**Authors:** Dongwook Oh, Myung-Hwan Kim, Tae Jun Song, Charles J. Cho, Kwangwoo Nam, Min Keun Cho, Joo Hyun Chun, Kyoungwon Jung, Kyu-pyo Kim, Jae Wha Kim

**Affiliations:** a Division of Gastroenterology, Department of Internal Medicine, University of Ulsan College of Medicine, Asan Medical Center, Seoul, Korea; b Division of Oncology, Department of Internal Medicine, University of Ulsan College of Medicine, Asan Medical Center, Seoul, Korea; c Biomedical Translational Research Center, Korea Research Institute of Bioscience and Biotechnology, Deajeon, Korea

**Keywords:** Pancreatic cancer, Chemotherapy-induced neutropenia, Gemcitabine, G-CSF, PLAG

## Abstract

**Background:**

Chemotherapy-induced neutropenia (CIN) may compromise planned chemotherapy, resulting in severe infection, dose reduction or delayed treatment. Orally administered 1-pamitoyl-2-linoleoyl-3-acetyl-rac-glycerol (PLAG) is a synthetic monoacetyldiglyceride, a product found in the antlers of sika deer. The aim of this study was to evaluate the effectiveness of PLAG for the prevention of CIN.

**Methods:**

A total of 48 patients with unresectable pancreatic cancer received gemcitabine-based palliative chemotherapy. Among those patients, 16 patients received PLAG (500 mg) twice daily from the start of chemotherapy to the completion.

**Results:**

The PLAG group showed a significantly lower incidence of neutropenia (absolute neutrophil count < 1,500 cells/mm^3^, grade 2-4), as compared to the control group (37.5% vs. 81.3%, P < 0.05). The absolute neutrophil counts (ANCs) of the PLAG group significantly less decreased from the baseline level compared to those of the control group (P < 0.05) and this significant difference in the reduction percentage of ANCs between the two groups was sustained throughout the courses of chemotherapy. No adverse events related to PLAG were observed.

**Conclusions:**

PLAG was shown to be clinically effective and safe in reducing the incidence of CIN in pancreatic cancer patients receiving gemcitabine-based chemotherapy.

## Introduction

Pancreatic cancer is one of the leading causes of death among gastrointestinal malignancies in the United States and Europe [1, 2]. Gemcitabine has been the standard first-line therapy for unresectable pancreatic cancer [3]. As the survival benefit of gemcitabine monotherapy is modest, however, various drugs have been investigated in combination with gemcitabine [4-9]. Gemcitabine with erlotinib combination chemotherapy showed significant survival benefits over gemcitabine monotherapy [7-9]. Myelosuppression, in particular neutropenia, is not uncommon during gemcitabine-based chemotherapy [9].

Severe neutropenia may be associated with life-threatening infections [10]. It also results in delays of the next cycle chemotherapy and/or dose reductions, leading to suboptimal chemotherapy delivery that may affect treatment outcomes [10, 11]. There is a need for hematopoietic stimulating agents for use in the prevention and/or recovery of neutropenia during cytotoxic chemotherapy. In current practice, long-acting G-CSFs have been used for the prevention of chemotherapy-induced neutropenia (CIN) [12]. However, these agents are administered parenterally and have considerable side effects.

**Figure 1:**
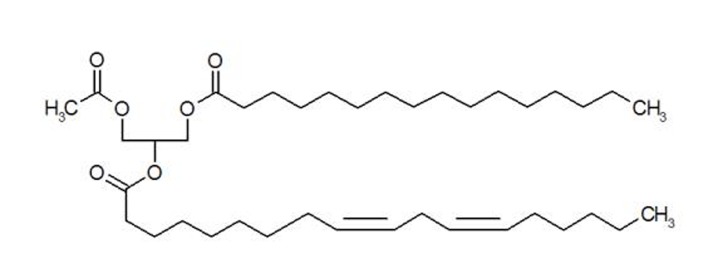
Structure of synthetic PLAG.

1-Pamitoyl-2-linoleoyl-3-acetyl-rac-glycerol (PLAG) is an orally available synthetic monoacetyldiglyceride that has been isolated from the antlers of sika deer (Cervus nippon Temminck) (Fig. 1) [13]. Deer antler is a traditional Asian medicine, prepared by drying the uncornified antler of a deer. It has been known that chemically synthetic PLAG can stimulate the proliferation of hematopoietic stem cells, bone marrow stromal cells, immune system cells including T and B lymphocytes, dendritic cells and macrophages, both *in vivo* and *in vitro* [13, 14].

Here we evaluate the effectiveness of synthetic PLAG for the prevention of CIN in patients with unresectable pancreatic cancer undergoing gemcitabine-based chemotherapy.

## Materials and Methods

### Patients

From January 2014 to September 2014, 16 patients with histologically or cytologically confirmed unresectable pancreatic cancer were enrolled in this study. Eligible patients had 1) locally advanced or metastatic cancer; 2) an age of ≥ 18 years; 3) an Eastern Cooperative Oncology Group (ECOG) performance status of ≤ 1; 4) adequate bone marrow function (absolute neutrophil count (ANC) ≥ 1,500/mm^3^, platelet count ≥ 10^5^/mm^3^); 5) normal renal (creatinine clearance ≥ 50 mL/min) and hepatic function (alanine aminotransferase and total bilirubin ≥ 2 times the upper limit of normal).

Historical controls were also recruited from Asan Medical Center from March 2012 to December 2013. The eligibility criteria for the control group were the same as those for cases who intake PLAG during gemcitabine-based chemotherapy. The control group (n = 32) was matched to the PLAG group (n = 16) based on age, performance status, chemotherapy cycle, comorbidity and disease extent. This study was approved by our hospital institutional review board.

### Study design and treatment protocol

All patients received gemcitabine 1,000 mg/m^2^ on days 1, 8, and 15 of each 4-week schedule and daily erlotinib at 100 mg orally. In the PLAG group, PLAG 500 mg was orally administered twice daily from the start of the chemotherapy to the completion. Hematology and serum chemistry analyses were performed at screening baseline, then weekly until the end of the study. Febrile neutropenia (FN) was defined as an ANC of less than 1,000/mm^3^ and an oral temperature of more than 38 °C on the same day or the following day after chemotherapy. If, on the day of chemotherapy administration, a patient’s ANC was reduced to 500 - 1,000/mm^3^ or if the absolute platelet count was reduced to 50,000 - 100,000/mm^3^, the gemcitabine dose was reduced by 75%. Gemcitabine was omitted for 1 week if the neutrophil count was lower than 500/mm^3^ or the absolute platelet count was lower than 50,000/mm^3^. Chemotherapy was discontinued if disease progression was observed in a follow-up CT scan, which was performed within 2 or 3 months after the initiation of chemotherapy. Erlotinib dose was interrupted in patients within tolerable rash and was reduced or discontinued if symptoms persisted for 10 - 14 days. Erlotinib dose was reduced for grade 2 diarrhea persisting for 48 - 72 h and for grade 3 diarrhea following resolution to grade 1; erlotinib was permanently discontinued for grade 4 diarrhea. Treatment continued until disease progression, unacceptable toxicity, withdrawal of patient’s consent or physician’s decision. Safety was evaluated throughout the entire study. Toxicity was graded based on the NCI Common Terminology Criteria for Adverse Events (CTCAE) version 3.0.

### Statistical analysis

The primary endpoint was neutropenia and the secondary endpoint was a safety profile. All analyses were performed using SPSS version 17.0 (SPSS Inc., Chicago, IL, USA). Descriptive statistics were used to evaluate demographics, and safety data continuous variables were compared using the Mann-Whitney U test, paired *t*-test, and independent T test. A P value of < 0.05 was considered statistically significant.

## Results

The baseline characteristics of patients are summarized in Table 1 and clinical outcomes are presented in Table 2. Six patients (37.5%) had locally advanced pancreatic cancer and the rest of the 16 patients (62.5%) had metastatic pancreatic cancer. The median number of treatment cycles administered was 2.5 (range 2 - 3). There were no significant differences between the PLAG group and the control group with respect to age, gender, disease extent, chemotherapy cycle, and ECOG performance.

**Table 1:** Baseline Characteristics of Patients

	PLAG group (N = 16)	Control group (N = 32)
Male/female	7:9	23:9
Median age (years, range)	56.5 (44 - 72)	59 (44 - 69)
Chemotherapy cycle		
Two cycles	8	16
Three cycles	8	16
Cancer stage		
Locally advanced	6	12
Metastatic	10	20
ECOG performance		
Grade 1	16	32

**Table 2:** Clinical Outcomes of Patients

Absolute neutrophil count (ANC) or %	PLAG group (N = 16)	Control group (N = 32)	P-value
ANC < 1,500 (grade 2 or higher)	6 (37.5%)	26 (81.3%)	P < 0.05
Depth of the ANC nadir > 50% of baseline level	6 (37.5%)	29 (90.6%)	P < 0.05
Depth of the ANC nadir > 75% of baseline level	0	12 (37.5%)	P < 0.05

**Figure 2:**
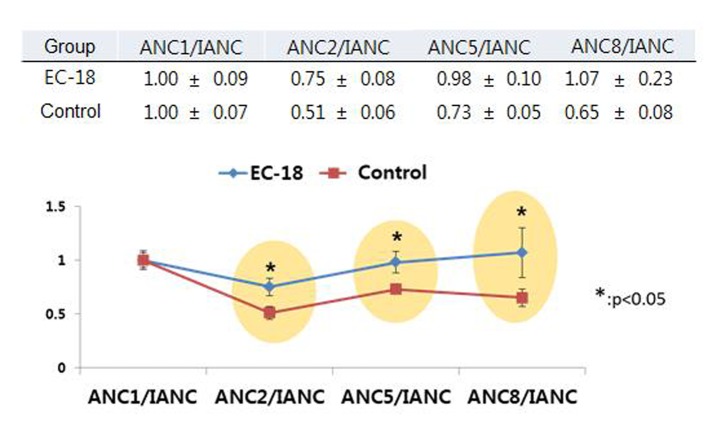
Trends in ANCs over the courses of chemotherapy between PLAG and control groups. The ANCs of PLAG group significantly less decreased from the baseline level compared to those of control group and this significant difference in reduction percentage of ANCs between the two groups was sustained throughout the courses of chemotherapy. Mean ± SE: mean ± standard error of mean. ANC: absolute neutrophil count; IANC: baseline neutrophil count prior to chemotherapy; ANC1: ANC obtained just prior to second time gemcitabine injection; ANC2: ANC obtained just prior to third time gemcitabine injection; ANC5: ANC obtained just prior to sixth time gemcitabine injection; ANC 8: ANC obtained just prior to ninth time gemcitabine injection.

**Figure 3:**
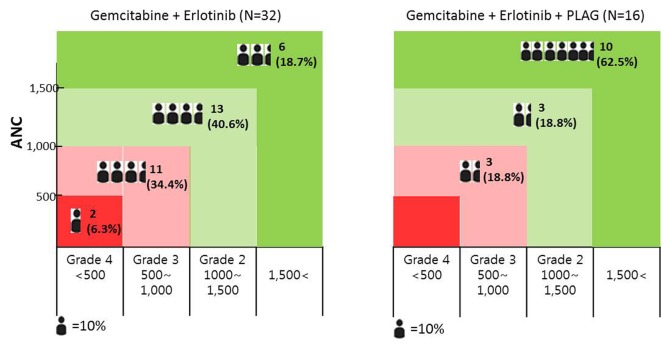
The distribution of chemotherapy-induced neutropenic patients according to the degree of neutropenia; the incidence of grade 2-4 neutropenia was significantly lower among patients who received PLAG compared to control group. Grade 4 neutropenia developed only in the control group.

The incidence of neutropenia (ANC < 1,500/mm^3^, grade 2-4) was significantly lower among patients who received PLAG, compared to the control group (37.5% vs. 81.3%, P < 0.05). By cycle, the reduction percentage of ANC was evaluated in both groups. At the baseline evaluation, the ANC did not differ between the two groups. However, the ANCs of the PLAG group significantly less decreased from the baseline level compared to those of the control group (P < 0.05) and this significant difference in the reduction percentage of ANCs between the two groups was sustained throughout the courses of chemotherapy (Fig. 2). Severe neutropenia (ANC < 500/mm^3^, grade 4) developed only in the control group (Fig. 3). The ANC nadir of the control group was significantly deeper than that of the PLAG group; a depth of ANC nadir of > 50% or 75% of the baseline level, respectively, was more frequently observed in the control group (Table 2, P < 0.05). FN did not occur in both groups.

PLAG was well tolerated in PLAG group. All patients completed intake of PLAG during the study period. There were no adverse events related to PLAG during chemotherapy including nausea/vomiting, bone pain, fatigue, and liver dysfunction.

## Discussion

This study was focused on the incidence of CIN for assessing the preventive effect of orally administered PLAG in patients who receive gemcitabine-based chemotherapy. PLAG administered orally during the courses of chemotherapy significantly reduced grade 2-4 neutropenia which may need chemotherapy dose modifications (dose delay/reduction) as compared with the control group (Table 2). In addition, severe neutropenia (ANC < 500/mm^3^) developed only in the control group (Fig. 3). In our study, FN did not develop in both groups, probably because gemcitabine is a chemotherapy regimen associated with a low risk for FN [12].

G-CSF is a recombinant growth factor that decreases the incidence and duration of severe neutropenia and minimizes infections as manifested by FN by stimulating the proliferation, differentiation, and activation of the neutrophil lineage, thereby reducing the neutrophil maturation time [12, 15-17]. Recently, pegylated G-CSF (pegfilgrastim) has been used for the prevention of CIN. Compared with original G-CSF (filgrastim), pegfilgrastim has a longer-acting effect equivalent to 10 - 11 days of filgrastim [18]. The administration of G-CSF within 24 h before or after chemotherapy is not recommended because of the theoretical potential for increasing chemotherapy toxicity to myeloid progenitor cells after growth factor stimulation (Table 3) [19]. Current guidelines recommend the use of prophylactic G-CSFs when the chemotherapy regimen is associated with a high risk (> 20%) for FN [12, 15-17]. In patients who receive a chemotherapy regimen associated with an intermediate risk (10-20%) for FN, however, long-acting G-CSFs can be used when patients have additional patient-related risk factors for FN such as old age or comorbidity [15-17].

**Table 3:** Comparison of PLAG and Long-Acting G-CSF for the Prevention of Chemotherapy-Induced Neutropenia

	Long-acting G-CSF (pegfilgrastim)	PLAG
Route of administration	Parenteral (subcutaneous injection)	Per oral
Administration mode	Once per chemotherapy cycle	Daily administration continuing throughout the course of chemotherapy
Timing of starting administration	Recommended to be administered 24 h after chemotherapy completion	Can be safely administered prior to or simultaneously with the initiation of chemotherapy
Side effects	Bone pain, fatigue, nausea, headache, splenic rupture, etc.	Not observed

The administration of G-CSF can cause various adverse events in patients who receive chemotherapy. Injection-site discomfort is common with G-CSF because it is administered by subcutaneous injection. Constitutional symptoms, such as fever, malaise, and influenza-like symptoms, are commonly developed after G-CSF administration. Bone pain, the most common side effect, develops in 10-30% of patients [15]. Serious adverse events such as splenic rupture, acute respiratory distress syndrome, though rare, can occur in patients receiving G-CSF [20-23]. In our study, side effects such as bone pain, fatigue, nausea, headache, or splenic rupture, which has been reported in the use of G-CSF, were not observed in any patients receiving PLAG.

In a cost-analysis of G-CSF, previous studies seem to heavily concentrate on medical costs related to FN and its consequences such as the incidence of FN, rate of hospitalization, IV antibiotic use and early mortality. However, “afebrile” neutropenia (ANC < 1,500/mm^3^, grade 2-4) may lead to dose delays or dose reductions as well as interference with the delivery of the full doses of the chemotherapy on time [24]. The resulting reduced dose intensity may worsen outcomes, especially in the setting of curative/adjuvant chemotherapy [25]. It is unlikely that much consideration was given to the impact of afebrile neutropenia on the relative dose intensity of the treatment received. This is because FN has an immediate impact on both mortality and the cost of treatment, whereas survival outcome is such a distant end point which needs long-term analysis [26]. Cost issues are likely to play a major role in limiting the use of long-acting G-CSF. If orally available PLAG with proven safety and effectiveness is much cheaper than long-acting G-CSF, PLAG may replace pegfilgrastim for the prevention of CIN in real world. This is because long-acting G-CSF (including biosimilar) is expensive, parenterally administered, and has considerable adverse events.

Due to the inherent limitation of a retrospective design and small-population of the current study, the effect of PLAG in the present study may become more evident in the large-scale prospective randomized placebo-controlled studies. Further studies are warranted to verify the effectiveness of PLAG in terms of FN prevention in patients receiving chemotherapeutic agent with higher myelosuppressive potency.

In conclusion, PLAG was shown clinically to be effective and safe in reducing the incidence of CIN in pancreatic cancer patients receiving gemcitabine-based chemotherapy.
